# Supplementary material for: The interactome of intact mitochondria by cross-linking mass spectrometry provides evidence for coexisting respiratory supercomplexes
Source: Mol Cell Proteomics. 2017 Dec 8;17(2):216–32. doi: 10.1074/mcp.RA117.000470 (PMC5795388; doi:10.1074/mcp.RA117.000470)
Supplement: Supplemental Data [file supp_RA117.000470_133922_0_supp_23978_fzffwf.zip › spectra_annotation/mito_DR_spectra_annotation/350-1-10-1-3-1.pdf]

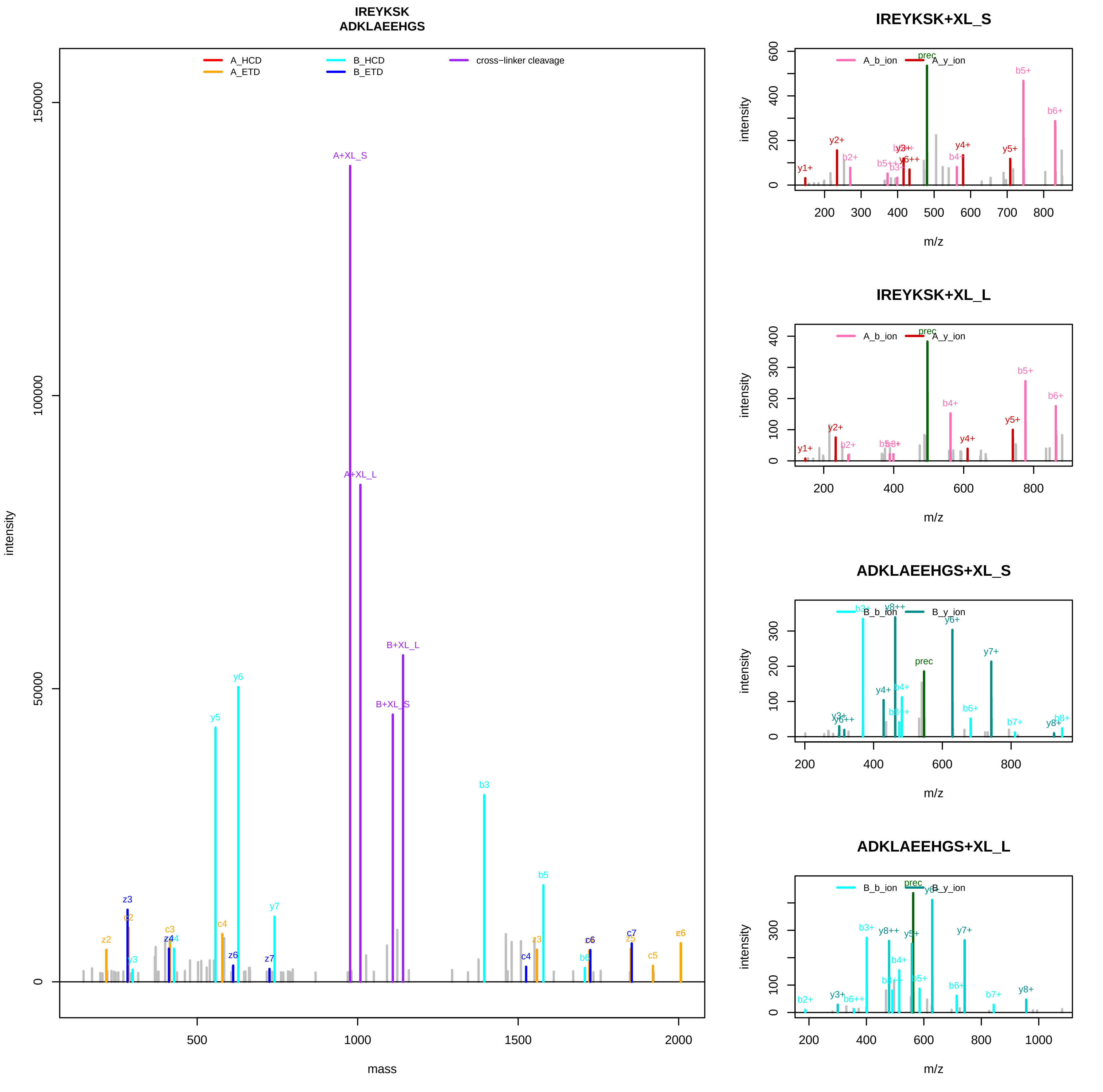

IREYKSK
ADKLAEEHGS
A_HCD
A_ETD
B_HCD
B_ETD
cross−linker cleavage
intensity
mass
0
50000
100000
150000
500
1000
1500
2000
A+XL_S
A+XL_L
B+XL_L
B+XL_S
y5
y6
y7
b3
b5
z2
z3
c2
c3
z4
c4
z6
z7
y3
c4
b6
c6
c7
z5
c5
z6
IREYKSK+XL_S
A_b_ion
A_y_ion
prec
y1+
y2+
b2+
b5++
b3+
y3+
y6++
b4+
y4+
y5+
b5+
b6+
m/z
IREYKSK+XL_L
ADKLAEEHGS+XL_S
B_b_ion
B_y_ion
b3+
y8++
y6+
y7+
y4+
b4+
b8++
b6+
b7+
y8+
b8+
y3+
y6++
ADKLAEEHGS+XL_L
b2+
y3+
b6++
b3+
y8++
y5+
b4+
b8++
b5+
b6+
y7+
b7+
